# Nitrate dynamics in natural plants: insights based on the concentration and natural isotope abundances of tissue nitrate

**DOI:** 10.3389/fpls.2014.00355

**Published:** 2014-07-23

**Authors:** Xue-Yan Liu, Keisuke Koba, Akiko Makabe, Cong-Qiang Liu

**Affiliations:** ^1^State Key Laboratory of Environmental Geochemistry, Institute of Geochemistry, Chinese Academy of SciencesGuiyang, China; ^2^Department of Environmental Science on Biosphere, Institute of Agriculture, Tokyo University of Agriculture and TechnologyFuchu, Japan

**Keywords:** atmospheric nitrate, denitrifier method, isotopic enrichment, isotopic fractionation, nitrate reductase, oxygen isotope, plant nitrate, soil nitrogen availability

## Abstract

The dynamics of nitrate (NO^−^_3_), a major nitrogen (N) source for natural plants, has been studied mostly through experimental N addition, enzymatic assay, isotope labeling, and genetic expression. However, artificial N supply may not reasonably reflect the N strategies in natural plants because NO^−^_3_ uptake and reduction may vary with external N availability. Due to abrupt application and short operation time, field N addition, and isotopic labeling hinder the elucidation of *in situ* NO^−^_3_-use mechanisms. The concentration and natural isotopes of tissue NO^−^_3_ can offer insights into the plant NO^−^_3_ sources and dynamics in a natural context. Furthermore, they facilitate the exploration of plant NO^−^_3_ utilization and its interaction with N pollution and ecosystem N cycles without disturbing the N pools. The present study was conducted to review the application of the denitrifier method for concentration and isotope analyses of NO^−^_3_ in plants. Moreover, this study highlights the utility and advantages of these parameters in interpreting NO^−^_3_ sources and dynamics in natural plants. We summarize the major sources and reduction processes of NO^−^_3_ in plants, and discuss the implications of NO^−^_3_ concentration in plant tissues based on existing data. Particular emphasis was laid on the regulation of soil NO^−^_3_ and plant ecophysiological functions in interspecific and intra-plant NO^−^_3_ variations. We introduce N and O isotope systematics of NO^−^_3_ in plants and discuss the principles and feasibilities of using isotopic enrichment and fractionation factors; the correlation between concentration and isotopes (N and O isotopes: δ^18^O and Δ^17^O); and isotope mass-balance calculations to constrain sources and reduction of NO^−^_3_ in possible scenarios for natural plants are deliberated. Finally, we offer a preliminary framework of intraplant δ^18^O-NO^−^_3_ variation, and summarize the uncertainties in using tissue NO^−^_3_ parameters to interpret plant NO^−^_3_ utilization.

## Plant nitrate (NO^−^_3_) in a natural context

Nitrogen (N) is a key factor in the control of the primary productivity in terrestrial plant ecosystems (Vitousek and Howarth, 1991; LeBauer and Treseder, 2008). Among the N species available to plants, ammonium (NH^+^_4_) is dominant in the inorganic N of unfertilized soils (Schimel and Bennett, 2004) and atmospheric N deposition (Stevens et al., 2011). Some plants prefer NH^+^_4_ (Britto and Kronzucker, 2013) while the roots of a few plants directly absorb organic N (Chapin et al., 1993; Näsholm et al., 2009; Hill et al., 2013). However, nitrate (NO^−^_3_) is an important N source for all plants because of its versatile functions in both plant nutrition and physiological regulations (Raven, 2003; Wang et al., 2012). The utilization of NO^−^_3_ (mainly uptake and reduction/assimilation) has been investigated intensively in plants through characterization of related enzymes including nitrate reductase (NR) and nitrite reductase (NiR) and their activities (NRA and NiRA, respectively) in response to different environmental conditions (Beevers and Hageman, 1969; Atkin et al., 1993; Kronzucker et al., 1995; Campbell, 1999). The framework of plant NO^−^_3_ studies has expanded in the past few decades due to the availability of molecular techniques. A few model plants have been used in understanding the transporters responsible for NO^−^_3_ uptake and transportation (Wang et al., 2012). Besides its function in nutrient supply, plant NO^−^_3_ and its metabolism contain unique information related to the mediation of plant physiology, diversity, and the ecosystem N cycle (Crawford, 1995; Tischner, 2000). However, evolution has yielded diverse strategies by which plants acquire N and NO^−^_3_ from natural environments to adapt to changes in ecosystem N availability (Chapin, 1980; Raven and Yin, 1998; Nacry et al., 2013). Therefore, there are considerable uncertainties in assessing the utilization of NO^−^_3_ by plants in natural habitats, which cannot be explained fully by laboratory-based mechanisms because of methodological constraints. Consequently, a great need exists for a straightforward estimation of plant NO^−^_3_ availability and a mechanistic understanding of the processes controlling plant NO^−^_3_ uptake and reduction. These can enhance our understanding of the role of plant NO^−^_3_ utilization in the ecosystem N cycle and the changes of plant growth and diversity with ecosystem N status (Lambers et al., 2008; Bloom et al., 2010; Boudsocq et al., 2012).

## Denitrifier method for NO^−^_3_ in natural plants

Natural abundance of stable isotopes in natural plants can integrate the information related to N sources and physiological processes (Högberg, 1997; Robinson, 2001; Craine et al., 2009). The stable isotopes include δ^15^N, δ^18^O, and δ^17^O for NO^−^_3_; ^15^N:^14^N, ^18^O:^16^O, and ^17^O:^16^O ratios expressed relative to atmospheric N_2_ and standardized mean ocean water (VSMOW), respectively (Coplen, 2011). These isotopes have been broadly used for studying plant N strategies and enzymatic dynamics in natural settings (Evans, 2001; Tcherkez and Farquhar, 2006; Granger et al., 2010). Nevertheless, it is difficult to measure the concentration and isotopes (δ^15^N and δ^18^O) of NO^−^_3_ in plant tissues precisely using traditional methods (Liu et al., 2012a). The use of the denitrifier method for measuring low (sub-nanomole) concentrations of NO^−^_3_ ([NO^−^_3_]) started during the mid-1980s (Lensi et al., 1985). The method has high sensitivity and is especially applicable for samples with low [NO^−^_3_] but with high dissolved organic carbon (DOC) (Christensen and Tiedje, 1988; Binnerup and Sørensen, 1992; Aakra et al., 2000). The denitrifier method developed for both δ^15^N and δ^18^O analysis is based on the isotopic analysis of nitrous oxide (N_2_O). The N_2_O is converted from sample NO^−^_3_ by cultured denitrifying bacteria (*Pseudomonas aureofaciens*; ATCC 13985) that lack N_2_O reductase activity (Sigman et al., 2001; Casciotti et al., 2002). The method was initially performed on seawater with 20–50 nmol NO^−^_3_. Since then, the application has been expanded widely to accommodate isotopic analysis of NO^−^_3_ in fresh water (e.g., groundwater, stream water, precipitation), soil and sediment water, soil extracts, as well as dissolved organic N (DON) in seawater and DON bound to diatoms as described by Koba et al. (2010a) and McIlvin and Casciotti (2011), respectively. This method has recently been used for measurements of NO^−^_3_ in natural plants and crops (Liu et al., 2012a, 2013a; Laursen et al., 2013; Bloom et al., 2014; Mihailova et al., 2014). The established protocol facilitates the Δ^17^O (Δ^17^O = [1 + δ^17^O] / [1 + δ^18^O]^0.5247^ − 1; Kaiser et al., 2007) analysis of leaf NO^−^_3_ to diagnose atmosphere-derived NO^−^_3_ in leaf uptake (Mukotaka, 2014).

**Figure 1:**
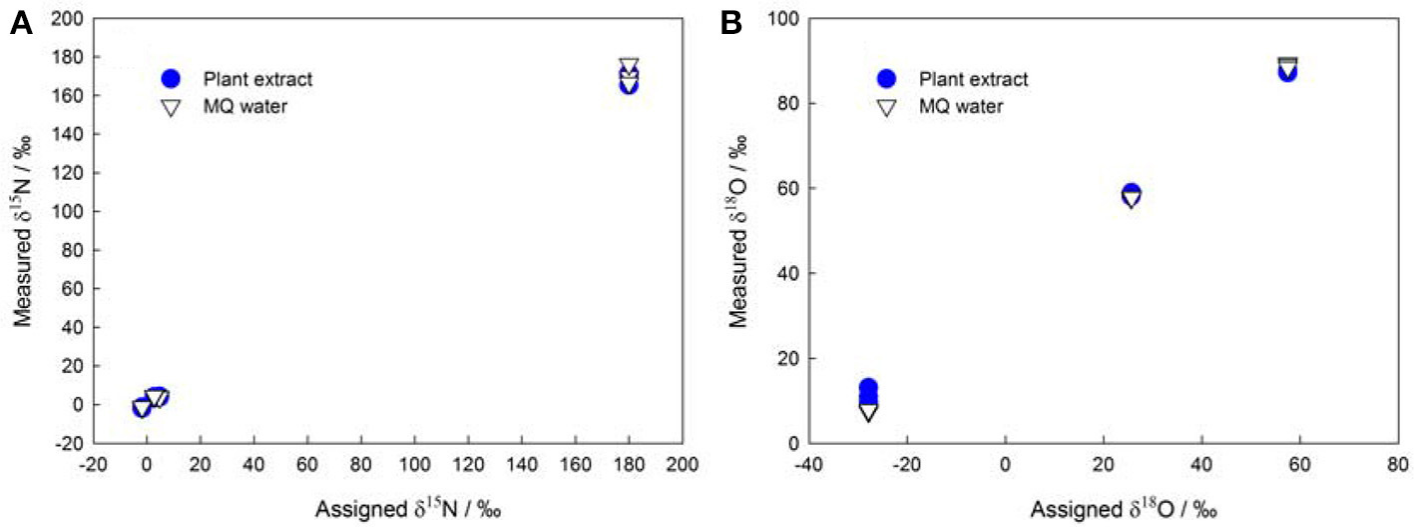
**Assigned isotopic ratios (**A**: δ^15^N; **B**: δ^18^O) of NO^−^_3_ standards (IAEA NO_3_, USGS-32, USGS-34, and USGS-35) shown against corresponding isotope values measured in MQ (Millipore) water and in plant extracts (the initial NO^−^_3_ in plant extracts was removed using the same protocol as that described in Liu et al., 2012a)**.

The denitrifier method enables more precise measurements of subnanomole amounts of NO^−^_3_ (Binnerup and Sørensen, 1992; Højberg et al., 1994) as compared to traditional methods that use flow injection analysis, ion chromatography, high-performance liquid chromatography, and Kjeldahl distillation. Thus, the denitrifier method overcomes the difficulties in determining NO^−^_3_ in plant, soil, and sediment samples (Norwitz and Keliher, 1986; Anderson and Case, 1999; Alves et al., 2000). Moreover, it greatly simplifies the pretreatment procedures and reduces the risk of contamination during plant NO^−^_3_ isotopic analysis (see the old δ^15^N protocol in Volk et al., 1979 and Evans et al., 1996). The denitrifier method especially avoids the influence of DOC in plant extracts (Haberhauer and Blochberger, 1999) on the δ^18^O of NO^−^_3_ (Figure 1) that was previously measured as carbon monoxide with TC/EA-IRMS (Michalski, 2010).

Compared with NRA assays, concentrations and isotopic signatures of tissue NO^−^_3_ provide more authentic evidence related to NO^−^_3_ uptake and reduction under *in situ* N availability. *In vitro* and *in vivo* NRA measurements (Stewart et al., 1992, 1993) do not reflect the *in situ* ability of plant NO^−^_3_ reduction. This is because firstly, the added amount of NO^−^_3_ (often at the micromolar level) during NRA assays is uniform. Moreover it is much higher than normal NO^−^_3_ availability and the endogenous NO^−^_3_ in natural plants. The synthesis of the NR enzyme or the activation of NRA, however, is substrate-inducible (Beevers and Hageman, 1969; Somers et al., 1983; Campbell, 1999). Secondly, the reagents used in the assay can affect the estimation of NRA. Different analytical settings (e.g., with or without ethanol) can alter the fluxes of NO^−^_3_ and photosynthate, resulting in different estimations (Ferrari and Varner, 1970; Aslam, 1981). Thirdly, NRA might be altered by pH adjustment and vacuum infiltration during the NRA analysis. High DOC concentrations in the plant extract also easily destroy the precision of the colorimetric determination of NO^−^_3_ or nitrite (NO^−^_2_) (Alves et al., 2000).

Since natural isotope analysis does not require artificial N addition, it presents no risk of changing the soil N pools and plant N-uptake kinetics (Liu et al., 2012b). The natural abundance approach does not disturb the N pools in plants and provides information related to the NO^−^_3_ behavior in plant tissues based on isotopic compositions and fractionations. In fact, the field application of ^15^NO^−^_3_ tracer is advantageous in terms of the total and short-term incorporation of NO^−^_3_ into plants (e.g., McKane et al., 2002; Wanek and Zotz, 2011). However, the added tracer cannot bypass the influence of soil microbial activity, which can greatly change the picture of N uptake and preference over time (Harrison et al., 2007). Measurements of cytosolic and vacuolar NO^−^_3_ concentrations have been conducted to explore factors controlling uptake, intracellular transport and assimilation. However, related techniques such as compartmental radiotracer (e.g., ^13^N; Kronzucker et al., 1995), efflux analysis, nuclear magnetic resonance, cell fractionation, and NO^−^_3_-selective microelectrodes showed high cost and low field operability (Zhen et al., 1991; Miller and Smith, 1996). The calculated [NO^−^_3_] is especially sensitive to the small error of the estimation of cytosolic and vacuolar volumes, the precisions of which are difficult to ascertain.

## Major sources and processes of NO^−^_3_ in natural plants

**Figure 2:**
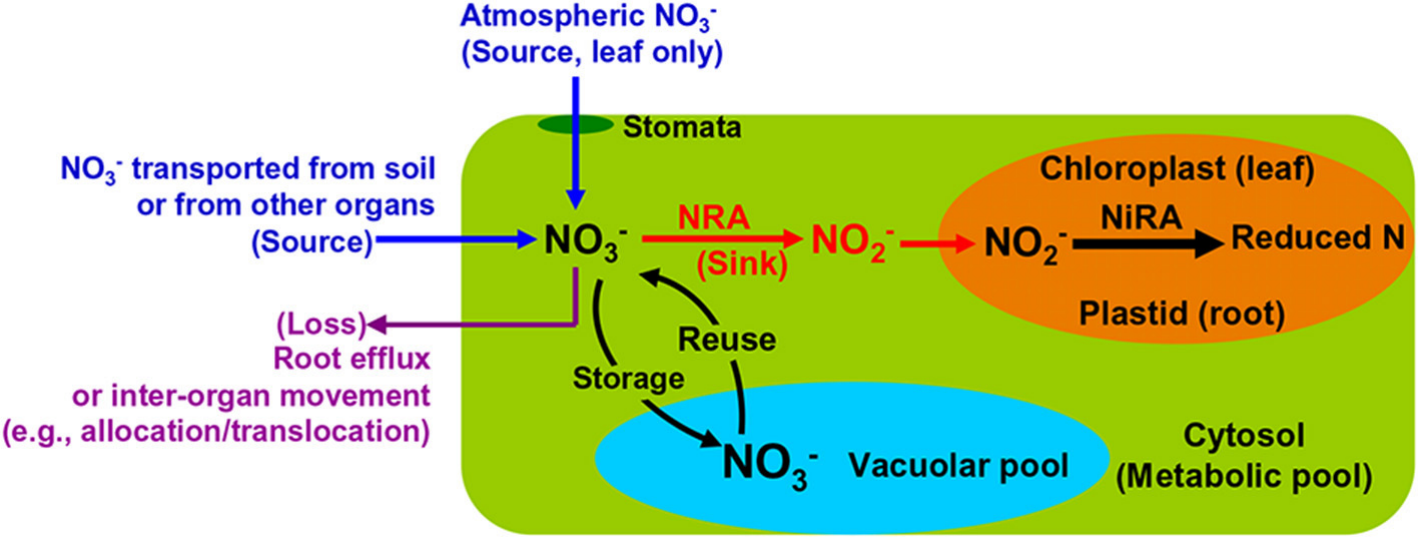
**Schematic map showing major NO^−^_3_ sources and processes in leaves and roots of natural plants**.

Root NO^−^_3_ uptake from the soil is achieved by active transportation (Wang et al., 2012). The extracellular NO^−^_3_ enters the cytosol of plant cells where it is either reduced by NR to NO^−^_2_ or stored in the vacuoles (Figure 2). The NO^−^_2_ will be transported into plastids (in root) or chloroplasts (in leaf) and reduced further by NiR to reduced N (Figure 2). Both NRA and NiRA are well known to be substrate-inducible, meaning that the *de novo* synthesis of the enzyme results from the presence and increase of the NO^−^_3_ in plants (Beevers and Hageman, 1969; Campbell, 1999). The induction of NRA by both soil and airborne NO^−^_3_ is an important mechanism to elucidate the interactions among NO^−^_3_ uptake, translocation/allocation, and reduction dynamics (Norby et al., 1989; Scheible et al., 1997a; Tischner, 2000).

The NO^−^_3_ transported by the xylem flow, either directly from soil or partially processed by root NR, is the initial NO^−^_3_ reaching leaves and shoots (Peuke et al., 2013). This is especially true for plants growing at some pristine sites (e.g., arctic tundra) where the atmospheric NO^−^_3_ availability is negligible. However, in regions with substantial NO^−^_3_ deposition, both atmospheric NO_x_ and NO^−^_3_ serve as potential sources of NO^−^_3_ in leaves (Wellburn, 1990; Raven and Yin, 1998; Sparks et al., 2001), especially for non-vascular plants such as mosses, which rely more on atmospheric nutrients (Liu et al., 2012c). Leaf NO^−^_3_ acquisition from the atmosphere is conducted through passive diffusion mechanisms wherein uptake through the stomata is dominant (Wellburn, 1990; Raven et al., 1992; Gessler et al., 2002) (Figure 2). The leaf-accessible NO^−^_3_ in the atmosphere includes an array of inorganic and organic ions and compounds (Wellburn, 1998; Teklemariam and Sparks, 2004; Vallano and Sparks, 2008). Although, previous tracer studies have described their incorporation into leaves (Hanson and Garten, 1992; Yoneyama et al., 2003; Lockwood et al., 2008), it is rather difficult to apply the natural abundance method for estimating field contributions of atmospheric NO^−^_3_. This can be attributed to the heterogeneity in chemical and deposition forms, and temporal and spatial distributions (Sievering et al., 2007; Sparks, 2009).

## Concentration levels and implications of NO^−^_3_ in natural plants

Nitrate cannot be produced in photoautotrophic plants, except in a few legumes (Hipkin et al., 2004). The presence of NO^−^_3_ in any part of a plant constitutes evidence of NO^−^_3_ uptake by the plant and reflects that external NO^−^_3_ is available; and that the rate of uptake is higher than the rate of reduction. The NO^−^_3_ that is extractable from a plant organ is often a sum of the amounts from the extracellular pool, cytosolic pool, and vacuolar pool (Figure 2). These pool sizes and turnover rates are regulated by both environmental and physiological factors (Zhen et al., 1991; Miller and Smith, 1996), which determine the isotopic signatures of the extracted NO^−^_3_. Generally, the concentration level and distribution of NO^−^_3_ in vascular plants and the variations among species is a complex result of two important factors: external availability (previously often evaluated through NO^−^_3_ concentration and net nitrification rate in soil) and physiological strategies (mainly including uptake, translocation, and reduction dynamics). Moreover, the external factors also consider the availability of NO^−^_3_ relative to NH^+^_4_ or other N sources because it can influence both plant NO^−^_3_ uptake and assimilation (Boudsocq et al., 2012; Liu et al., 2012c; Britto and Kronzucker, 2013) while the physiological factors include the affinity of plants to different soil NO^−^_3_ levels (Wang et al., 2012; Kalcsits and Guy, 2013).

**Figure 3:**
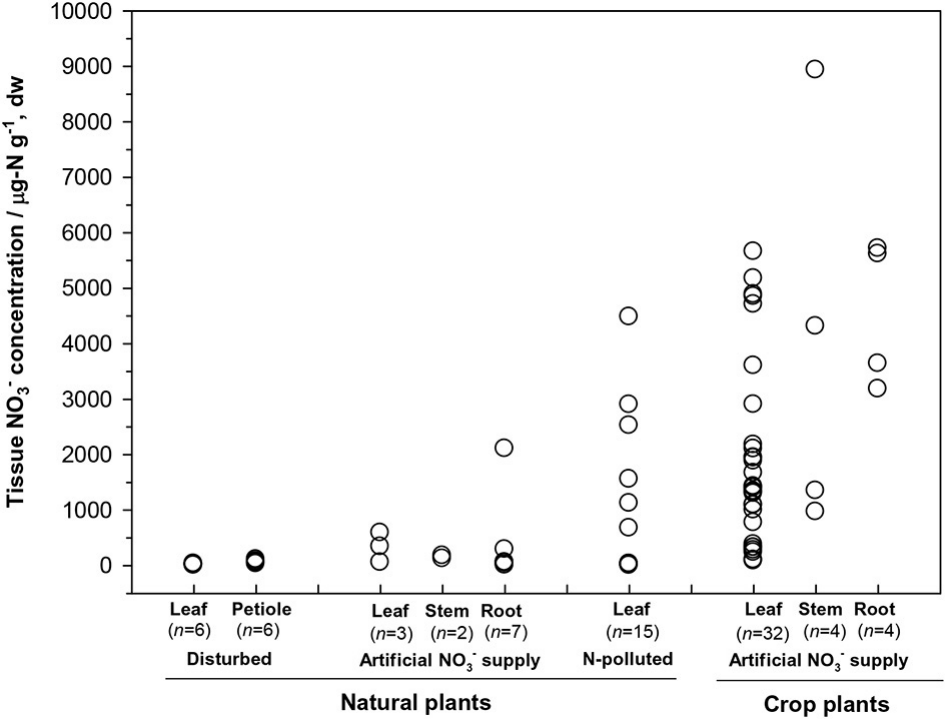
**Tissue NO^−^_3_ concentrations in natural plants growing under disturbed conditions (acidic irrigation and liming; Gebauer et al., 1988), in N-polluted forest plants (Stams and Schipholt, 1990), in natural and crop plants with artificial NO^−^_3_ supply (data of natural plants were cited from Gebauer et al., 1984; Stadler and Gebauer, 1992; Robe et al., 1994; Simon et al., 2014. Data of crop plants were cited from Evans et al., 1996; Yoneyama and Tanaka, 1999; Prasad and Chetty, 2008 and references cited therein)**.

**Figure 4:**
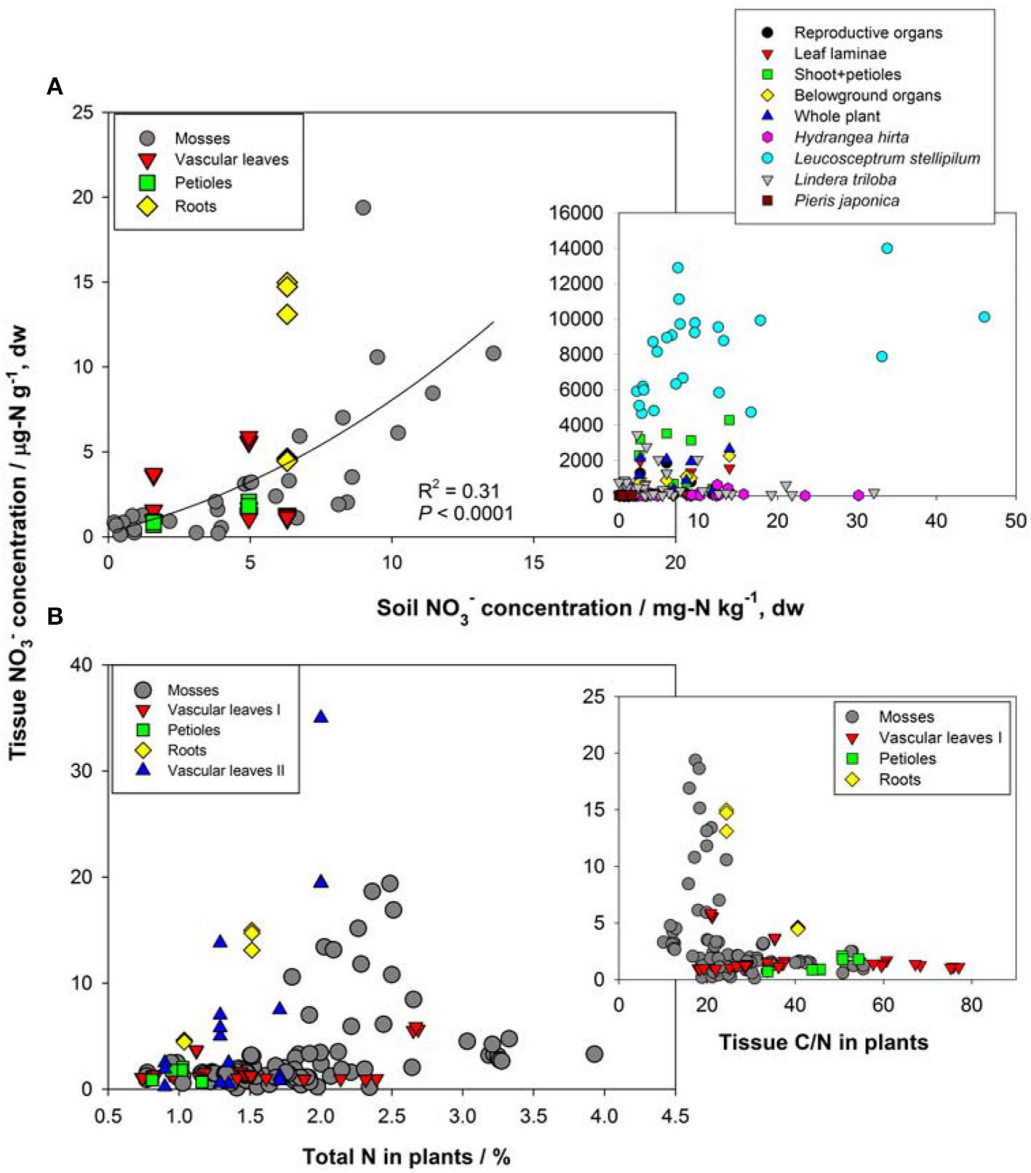
**(A)** Relation between NO^−^_3_ concentrations in soil and natural plants. Plant NO^−^_3_ data in the left panel are shown for individual samples in Guiyang, southwestern China and western Tokyo, Japan reported by Liu et al. (2012a, 2013a). Plant NO^−^_3_ data in the right panel show organ-specific and whole-plant concentrations (averages of different species) in ecosystems of Central Europe (see details in Gebauer et al., 1988), and leaf NO^−^_3_ of different species (*H. hirta, P. japonica, L. stellipilum, L. triloba*) in a temperate forest of central Japan (Koyama et al., 2013). **(B)** Relations between total N, C/N, and tissue NO^−^_3_ concentration in natural plants. Mosses include different species in different habitats of Guiyang, Southwestern China, and Western Tokyo, Japan (cited from Liu et al., 2012a,c). Vascular leaves I, petioles and roots were reported for a coniferous and a broadleaved plant in western Tokyo, Japan (cited from Liu et al., 2013a). Vascular leaves II included fern, oak, and pine species at the Camp Paivika and Camp Osceola forest sites in the San Bernardino Mountains of southern California, USA (cited from Fenn et al., 1996).

First, the distribution of organ-specific NO^−^_3_ concentrations among plants under different growing conditions (Figures 3, 4A) showed that plants growing in natural soils might also have a high NO^−^_3_ accumulation. In natural forests, leaf NO^−^_3_ concentrations of some species can be as high as 1000–10000 μ g-N g^−1^ dw (Figure 4A; Gebauer et al., 1988; Koyama et al., 2013), which was even higher than those of some crops (e.g., Bloom et al., 2014) and N-polluted natural plants (Figure 3). Plant NO^−^_3_ concentrations are indicators or predictors of the soil N cycle (e.g., soil nitrification and soil NO^−^_3_) and forest N pollution (Stams and Schipholt, 1990; Aber et al., 1998; Fenn and Poth, 1998; Koba et al., 2003). Such concentrations show higher sensitivities than bulk N and NRA parameters in revealing species-level responses to N enrichment (Fenn et al., 1996; Jones et al., 2008; Tang et al., 2012). The increase in NO^−^_3_ concentration in roots and or leaves with external NO^−^_3_ was observed under both natural soil conditions and experimental N addition (e.g., Stewart et al., 1993; Lexa and Cheeseman, 1997; Wang and Schjoerring, 2012). However, the level of leaf NO^−^_3_ and its response to soil NO^−^_3_ variation differ among species with distinct uptake or accumulation rates. For example, the NO^−^_3_ concentrations in plants (mostly as mosses) we recently investigated (Liu et al., 2012a,c, 2013a) were much lower than those reported by Gebauer et al. (1988) or Koyama et al. (2013) on vascular plants (Figure 4A) when compared within a similar soil [NO^−^_3_] range (e.g., 0–15 mg-N kg^−1^ soil, dw). Besides, the correlation between leaf NO^−^_3_ and soil NO^−^_3_ is apparent for plants with low NO^−^_3_ concentrations (Figure 4A). However, synthesis or extrapolation to different plants with distinct NO^−^_3_ accumulation abilities should be done carefully when evaluating soil N enrichment or N saturation.

Second, considerable differences (up to 4–5 orders) exist in the level of NO^−^_3_ among plant organs and species (Figures 3, 4A). The organ-specific patterns of NO^−^_3_ accumulation among coexisting plants can differ with soil N availability and the plant growing stage (Gebauer et al., 1984; Stewart et al., 1993; Liu et al., 2013a). However, this has complicated the use and selection of proper organs and species to evaluate ecosystem N availability based on tissue NO^−^_3_ analysis. McKane et al. (2002) used ^15^N tracers in the field to show that NO^−^_3_ uptake in the tundra plants did not passively follow external availability, but depended on specific ecophysiological traits. NO^−^_3_ preference in *Carex* was determined by the appearance of ^15^N tracer in *Carex* biomass, which showed that the NO^−^_3_ preference might reflect only the ^15^NO^−^_3_-acquiring efficiency associated with root traits, but not NO^−^_3_ assimilation given significantly lower NRA in *Carex* than in other species (Nadelhoffer et al., 1996). Therefore, additional studies should be conducted to determine the extent of organ-specific and species-specific variability of NO^−^_3_ concentration that reflects plant NO^−^_3_ strategy, and the heterogeneity of NO^−^_3_ available to roots. The available data for natural plants revealed a clear increase in NO^−^_3_ concentration with bulk N while a decrease with C/N (a clear turning at the C/N of 20–30) in different organs or tissue types (Figure 4B). Similarly, Zhen and Leigh (1990) reported that shoot NO^−^_3_ accumulated as a linear function of bulk N in wheat plants once a threshold N was exceeded. These results reflected the regulation of overall physiological N demand on the NO^−^_3_ utilization in natural plants (Imsande and Touraine, 1994). The regulation might be unidirectional because the contribution of NO^−^_3_ to bulk N assimilation appears to be much lower than that for other N forms in plants (portrayed in Figure 4B). The complexity of the mutual regulations behind the inverse relation between NO^−^_3_ and C/N might be comparable with the multi-scale inverse relation prevailing between NO^−^_3_ and organic C observed in different ecosystems (Taylor and Townsend, 2010). So far, little direct and simple evidence has been obtained for the driving mechanisms of C and N metabolism on NO^−^_3_ uptake, allocation, and accumulation in natural plants. A clearer relation is that even when external NO^−^_3_ is uniform, the NO^−^_3_ concentration is often higher in organs (especially for growing leaves) of species with higher NRA than in those with lower NRA (Gebauer et al., 1988; Cruz et al., 1991; Widmann et al., 1993; Min et al., 1998). Mutual induction between the maintenance of high NO^−^_3_ concentration and that of NR synthesis or NRA activation were elucidated in view of C metabolism and N demand in response to availability and growing conditions (Stewart et al., 1993; Scheible et al., 1997a,b; Scheurwater et al., 2002). The lower NO^−^_3_ concentration and NRA might be associated with lower N metabolism and demand in organs and plants with higher C/N and vice versa. Therefore, except regulation by soil NO^−^_3_ concentration, the uptake and distribution of NO^−^_3_ in a plant might follow the regime of organ-specific or whole-plant metabolic activities.

Other factors such as light and water regimes might also influence plant NO^−^_3_ accumulation through the pathway of photosynthetic regulation (Widmann et al., 1993; Simon et al., 2014). Cárdenas-Navarro et al. (1999) found concurrent and linearly correlated changes in whole-plant NO^−^_3_ and water content during the day–night cycle, reflecting a homoeostasis effect of endogenous NO^−^_3_ concentration. Besides, as discussed above, the heterogeneity of soil NO^−^_3_ available to roots of coexisting species should not be excluded considering the differences in root morphology and spatial distribution. Given the difficulties in determining rhizospheric soil NO^−^_3_ concentration and flux, it would be promising to measure NO^−^_3_ concentrations in roots to evaluate NO^−^_3_ availability to the whole plant or aboveground organs.

## Isotopic systematics of NO^−^_3_ in plants

Stable isotopes of NO^−^_3_ in plants are controlled mainly by NO^−^_3_ sources and isotopic effects involved in NO^−^_3_ acquisition and reduction processes (Robinson et al., 1998; Comstock, 2001; Evans, 2001; Cernusak et al., 2009).

**Figure 5:**
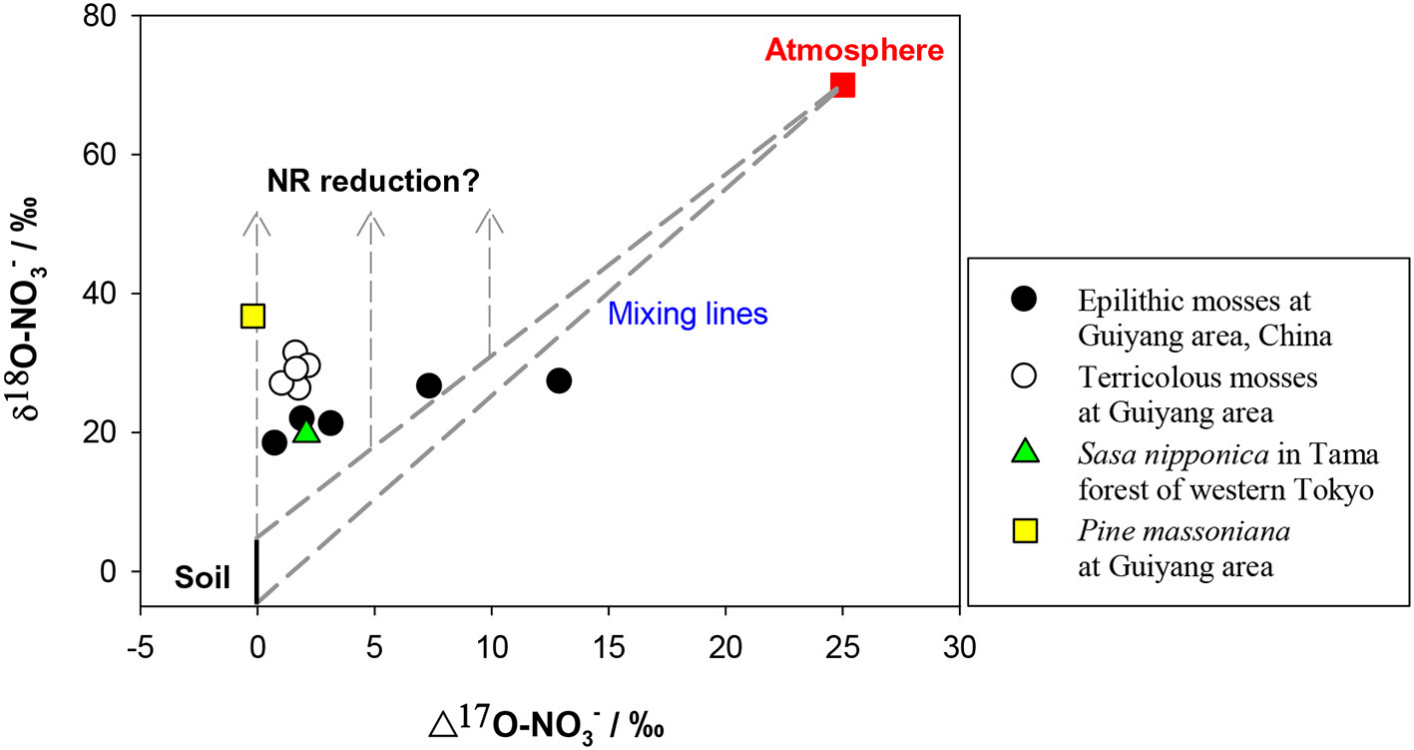
**Preliminary relation between δ^18^O and Δ^17^O values of NO^−^_3_ in mosses and vascular plants**. The δ^18^O and Δ^17^O values were considered respectively, as −5 to 5‰ and 0‰ for soil NO^−^_3_ (black and solid line), 70 and 25‰ for atmospheric NO^−^_3_ (red square). Dashed lines show the isotopic range of mixing between atmospheric and soil sources. Dashed lines with arrows show the vectors of δ^18^O enrichments because of NR reduction.

The δ^15^N of NO^−^_3_ in soil is reported mostly within −10 to +10‰ however, the δ^15^N of newly-produced NO^−^_3_ in soil is usually low because of strong isotopic effects of nitrification, on the other hand, the values can be elevated at sites with marked denitrification (Mariotti et al., 1981; Högberg, 1997; Koba et al., 1998, 2003, 2010b; Houlton et al., 2006; Takebayashi et al., 2010). Atmospheric NO^−^_3_ has a wider δ^15^N range (−15 – +15‰) because of its complex production pathways and sources (Heaton, 1990; Felix et al., 2012; Altieri et al., 2013). The δ^15^N of NO^−^_3_ is generally lower in wet than in dry deposition (Heaton et al., 1997; Elliott et al., 2009), but both often show a δ^15^N range overlapping with that of soil NO^−^_3_. The δ^18^O of initial NO^−^_3_ produced in soil is usually estimated using the δ^18^O of *in situ* H_2_O (normally −25 – 4‰) and atmospheric O_2_ (ca. 23.5‰) in a 2:1 ratio, assuming no exchange and fractionation of oxygen (O) isotopes occurs during nitrification and the NO^−^_3_ is produced solely through chemolithoautotrophic nitrification (Amberger and Schmidt, 1987). However, kinetic isotopic fractionation and O exchange between NO^−^_2_ and H_2_O often occur during nitrification, which can eliminate the isotopic signal of O_2_ effecting lower δ^18^O than the predicted values (Fang et al., 2012). The O of NO^−^_3_ in atmospheric deposition is derived mainly from O_2_ and O_3_, which have distinctly higher δ^18^O and Δ^17^O signatures than those of soil NO^−^_3_. In contrast to the overlapping δ^15^N for different NO^−^_3_ sources, δ^18^O and or Δ^17^O provide a clear separation between soil and atmospheric NO^−^_3_ sources. The δ^18^O of soil NO^−^_3_ produced by nitrification is distinctly lower (mean = −4.0‰ −7.3 to −0.9‰ Fang et al., 2012) than that of atmospheric NO^−^_3_ (60 − 100‰). The latter has high Δ^17^O values (around 25‰) in contrast to 0‰ of soil-derived NO^−^_3_ (Kendall et al., 2007; Michalski, 2010; Costa et al., 2011) (Figure 5).

The process of NO^−^_3_ entry into root cells and subsequent transport processes within plants *per se* cause no isotope effect because of the lack of bond breakage. However, the acquisition of NO^−^_3_ through mycorrhizae to root cells potentially causes an isotopic difference between tissue NO^−^_3_ in roots and NO^−^_3_ in soil. Root NO^−^_3_ may be enriched in heavier isotopes relative to soil NO^−^_3_ if the NO^−^_3_ has experienced reduction during the N assimilation of mycorrhizae associated with the roots. Mycorrhizal fungi have substantial NO^−^_3_ reduction capacity (Ho and Trappe, 1975), but the fungal NR is present only in the presence of NO^−^_3_ and absence of NH^+^_4_ (Cove, 1966). So far, the isotopic effect of NO^−^_3_ acquisition through mycorrhizae on tissue NO^−^_3_ in natural plants has not been estimated or differentiated. Pate et al. (1993) demonstrated that the bulk δ^15^N differences between non-mycorrhizal and mycorrhizal species (with significant NO^−^_3_ storage and NRA) reflected the utilization of different N sources. There appears to be little or no isotopic discrimination within the plant during or subsequent to uptake of NO^−^_3_. Mycorrhizal fungi are expected to show higher bulk δ^15^N than available N sources [potentially including NO^−^_3_, NH^+^_4_, and DON (at least amino acids)] in soil and bulk N of host plants. However, the isotopic mechanism differed from that of tissue NO^−^_3_ and the isotope effect differed among mycorrhizal types (Högberg, 1997; Craine et al., 2009; Hobbie and Högberg, 2012). Högberg et al. (1999) showed that the ECM fungus had higher bulk δ^15^N relative to the *Pinus sylvestris* plant, and the fractionation against ^15^N was smaller when NO^−^_3_ was the source than when NH^+^_4_. It caused a marginal decrease in δ^15^N of the N passing from the substrate through the fungus to the host, which is explained by the small size of the fungal N pool relative to the total N of the plant, i.e., the high efficiency of transfer (Emmerton et al., 2001; Hobbie and Högberg, 2012). The significant shift in δ^15^N of fungal species was a function of fungal physiology; thus, it is difficult to constrain the N sources (using bulk δ^15^N) by mycorrhizal fungi or their plant partners in natural conditions (Emmerton et al., 2001).

**Figure 6:**
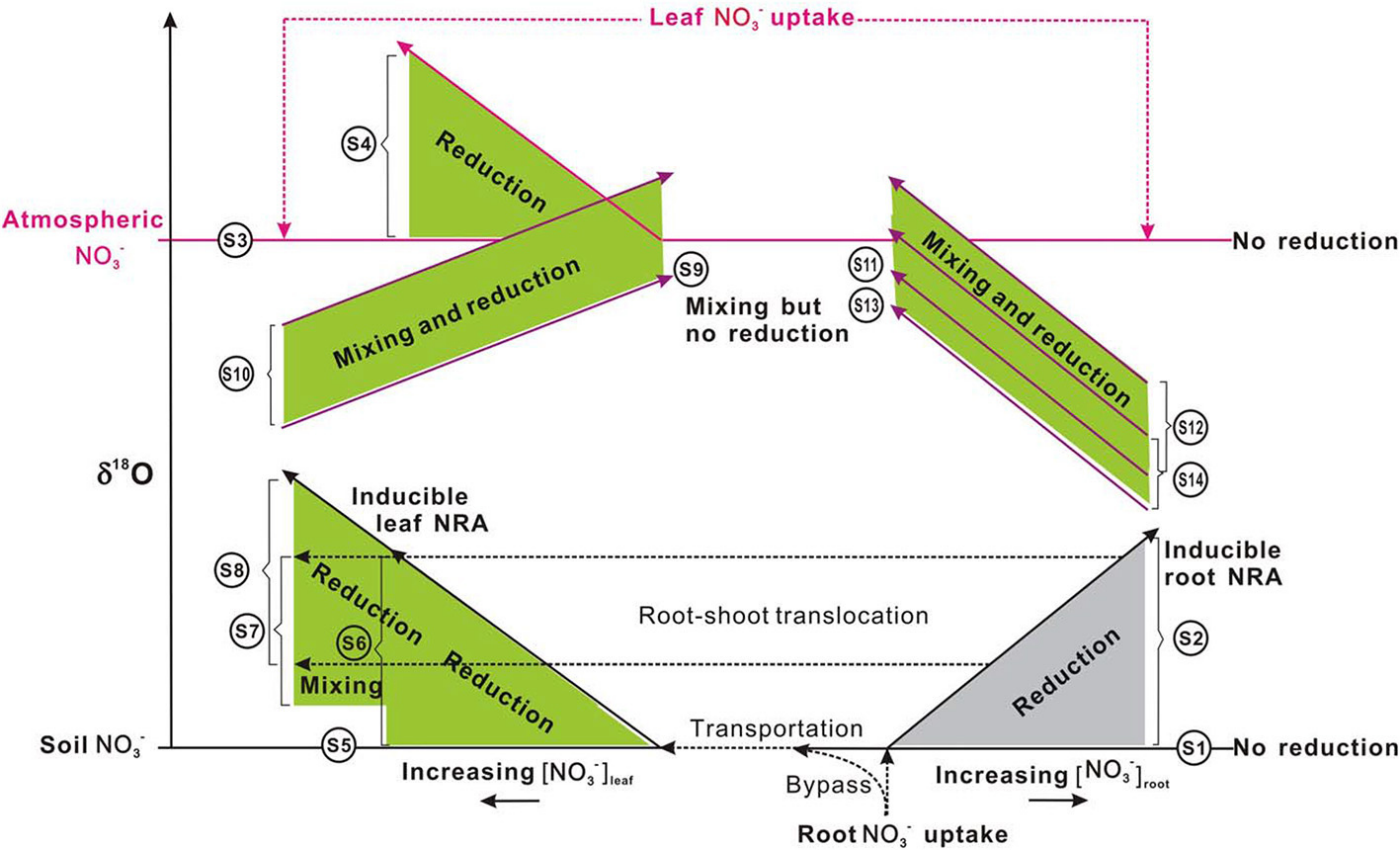
**Schematic showing δ^18^O-NO^−^_3_ variations in plants under different uptake (from soil and or atmospheric sources: distinct in the δ^18^O value), translocation (from soil and or root to shoot), and reduction (potentially inducible by increasing [NO^−^_3_] or no reduction and no isotopic enrichment with NO^−^_3_ accumulation, depending on species)**. Long and short solid lines with arrows respectively show the vectors of δ^18^O-NO^−^_3_ and [NO^−^_3_] variations. Dashed lines with arrows show the uptake, transportation, and translocation of NO^−^_3_ from the soil to roots and or to leaves, from atmosphere to leaves, during which isotope effects were regarded as negligible. Shaded areas (gray for roots, green for leaves) show isotopic enrichment during the mixing of different sources (the δ^18^O-NO^−^_3_ in plants should be distributed between the δ^18^O values of sources, depending on the fraction of each source) and or the occurrence of NR reduction activities (the δ^18^O-NO^−^_3_ in plants would be higher than the δ^18^O of sources but the magnitude of enrichment depends on *in situ* NR dynamics; presumably less than that presented in Table 1). For scenarios that occurred, leaf uptake of atmospheric NO^−^_3_ was assumed to be homogeneous. The shaded area, the spatial distance, and length of lines had no quantitative implications. S1–S12 correspond to scenarios 1–12 in the main text. Briefly, S1, no occurrence of NO^−^_3_ reduction in roots; S2, (inducible) root NO^−^_3_ reduction; S3, no NO^−^_3_ was transported from soil to leaves and leaf NO^−^_3_ was derived from the atmosphere, but no reduction occurred; S4, no NO^−^_3_ was transported from soil to leaves and leaf NO^−^_3_ was from atmosphere and (inducible) reduction occurred; S5, leaf NO^−^_3_ was taken up directly from the soil, but no reduction occurred; S6, leaf NO^−^_3_ was taken up from the soil and reduction occurred therein; S7, leaf NO^−^_3_ is completely or partially transported from the root where it has experienced reduction, but no further reduction in the leaf; S8, leaf NO^−^_3_ is completely or partially transported from the root where it has experienced reduction, and is further reduced in the leaf; S9, leaf NO^−^_3_ was from both atmosphere and soil but no reduction occurred in the leaf; S10, leaf NO^−^_3_ was from both atmosphere and soil, and reduction occurred in the leaf; S11, leaf NO^−^_3_ is a mixture of atm-NO^−^_3_ and root NO^−^_3_ but no reduction occurred; S12, leaf NO^−^_3_ is a mixture of atm-NO^−^_3_ and root NO^−^_3_, and reduction occurred in the leaf; S13, leaf NO^−^_3_ is a mixture of soil NO^−^_3_, atm-NO^−^_3_, and root NO^−^_3_, but no reduction occurred in the leaf; S14, leaf NO^−^_3_ is a mixture of soil NO^−^_3_, atm-NO^−^_3_, and root NO^−^_3_, and reduction occurred in the leaf. The δ^18^O differences between S13 and S11, between S12 and S14 depend on the fraction of soil NO^−^_3_ in the mixed pool of leaves.

The efflux of NO^−^_3_ from root to soil or the subsequent transport of NO^−^_3_ within plants is not expected to discriminate ^15^N as with the entry of soil NO^−^_3_ into root cells (Mariotti et al., 1982; Shearer et al., 1991). This can be attributed to that the diffusion of NO^−^_3_ through the membrane carriers of plant cells does not cause bonding breakage or consumption (Werner and Schmidt, 2002; Granger et al., 2004; Needoba et al., 2004). However, isotopic differences can occur between organs if partial NO^−^_3_ reduction occurs in roots before transportation. The transport of NR-processed NO^−^_3_ from roots to leaves might be misunderstood as isotopic fractionations of NO^−^_3_ transport or NO^−^_3_ reduction in shoots. So far, isotopic fractionations (ε = (^l^k/^h^k − 1) × 1000, where ^l^k and ^h^k respectively stand for the reaction rate constants for lighter and heavier isotopes) during the reduction of NO^−^_3_ by NR in leaves were reported as 15‰ for both N in spinach (Ledgard et al., 1985; Tcherkez and Farquhar, 2006) and O in wheat (Olleros-Izard, 1983) (Table 1). Direct measurement of endogenous NO^−^_3_ reduction in mosses after N deprivation showed similar values (Liu et al., 2012b) (Table 1). Although, NR isotopic fractionations have not been directly measured in roots, predictions can be made about the net enrichment of NO^−^_3_ isotopes in roots relative to those of soil NO^−^_3_ (Δ_root_; expressed as δ_root_ − δ_soil_). These values should be either negligible if substantial NO^−^_3_ reduction did not occur (Scenario 1; Δ_root_ = δ_root_ − δ_soil_ ≈ 0), or be close to the reported ε values of NRA in leaves (ε_NR_) (0 − 27‰ Table 1) if NO^−^_3_ reduction occurred in the root (Scenario 2; Δ_root_ = δ_root_ − δ_soil_ ≈ ε_NR_ > 0) (Figure 6). However, if the modification of soil NO^−^_3_ isotopes by soil microbial activities such as denitrification occurred later than root uptake, the observed isotopic values of root NO^−^_3_ can also be slightly lower than those of soil NO^−^_3_ despite reduction in roots (e.g., in the fine roots of a conifer investigated in Liu et al., 2013a). Furthermore, the variation of NO^−^_3_ isotopes with soil depth directly caused isotopic differences in initial NO^−^_3_ sources available to co-existing plants with different root depths. Therefore, considering this fact, soil reference samples should be collected corresponding to root distribution for characterizing the soil NO^−^_3_ isotopes available to specific plants.

**Table 1:** Isotopic effects reported for NO^−^_3_ reduction (^*^) or net NO^−^_3_ assimilation in different biota.

**Biota**	**15_ε_ / ‰**	**18_ε_ / ‰**	**References**
Eukaryotic NR enzymes (from fungus and marine diatoms)	26.6*	24.9*	Karsh et al., 2012
Moss	12.1*	14.4*	Liu et al., 2012b
Strains of prokaryotic plankton	0.4–8.6	0.9–8.1	Granger et al., 2010
Spinach and wheat	15.0*	15.0*	Olleros-Izard, 1983; Ledgard et al., 1985; Tcherkez and Farquhar, 2006
Eukaryotic algae	5.6–20.4	5.1–21.0	Granger et al., 2004
Marine phytoplankton	2.7–15.2	–	Needoba and Harrison, 2004
	4–9 (field) 2.2–6.2 (lab)	–	Needoba et al., 2003
Tomato	11.3–12.9	–	Evans et al., 1996
Leafy vegetable	14.2–18.1	–	Yoneyama and Kaneko, 1989; Yoneyama et al., 2003
Grasses	0.0–3.3	–	Mariotti et al., 1982
Pearl Millet and soybeans	0.0-9.5	–	Mariotti et al., 1980, 1982; Bergersen et al., 1988
Red clover	1.7–6.5	–	Kohl and Shearer, 1980

In a closed system, isotopic enrichment occurs with the enzymatic consumption of substrate NO^−^_3_ and ε_NR_ is expressed as Δ/ln[NO^−^_3_]_remaining_ fitted to the Rayleigh isotope fractionation model, where Δ represents the isotopic difference of remaining NO^−^_3_ from the initial NO^−^_3_ (δ_remaining_ − δ_initial_) (e.g., Granger et al., 2004, 2010). Isotopic enrichment also takes place for NO^−^_3_ remaining in plants after deprivation of NO^−^_3_ or N supply, because the tissue NO^−^_3_ pool is only changed by the NRA in a closed system (e.g., Liu et al., 2012b). Thus far, no experimental work has been done to explain the variability of ^18^ε_NR_ in and among vascular plants. In NO^−^_3_-supply studies, shoots tend to have higher δ^15^N values because of the allocation of root NR-processed NO^−^_3_ from roots to shoots (Kalcsits and Guy, 2013) or significantly higher ^15^ε_NR_ (by 3.3–6.9‰) than roots (Yoneyama and Kaneko, 1989; Evans et al., 1996; Yoneyama et al., 2001). Evidence from marine biota showed that both ^15^ε_NR_ and ^18^ε_NR_ can vary with growing conditions and that significantly different ε values exist among species (Table 1). In field conditions, NO^−^_3_ in an organ is more likely to be an open system with continuous source inputs (uptake), sinks (reduction), and outputs (translocation) (Figure 2). The uptake and allocation often occur according to the reduction ability and the distribution of NR, for example, a higher concentration and more NR are likely to exist in growing leaves (Gebauer et al., 1988; Cruz et al., 1991; Widmann et al., 1993). Passive or high accumulation as in mosses (Liu et al., 2012c) can happen in some organs such as conifer roots that are unable to reduce it (Liu et al., 2013a). Therefore, δ values of tissue NO^−^_3_ might not always follow the normal “Rayleigh type” relation, instead might increase with the increase in tissue [NO^−^_3_] or show a non-significant correlation with [NO^−^_3_] in the tissues (Liu et al., 2012c, 2013a). In fact, experimental studies have also shown the interplay of plant NO^−^_3_ uptake and reduction activity. The ^15^N discrimination during NO^−^_3_ assimilation in several higher plants was positively correlated with the supplied and tissue NO^−^_3_ concentrations, and negatively correlated with plant age (Kohl and Shearer, 1980; Mariotti et al., 1980, 1982; Bergersen et al., 1988; Liu et al., 2013a). Accordingly, the Rayleigh relation between NO^−^_3_ and its isotopes is not always applicable to examine ε_NR_ values and NO^−^_3_ reduction in organs of natural plants.

For some plants, NO^−^_3_ is not available in soil substrates. It can only be acquired from deposition (e.g., non-vascular plants or epiphytes). Alternatively, it is not available in deposition but can only be taken up from the soil (e.g., plants growing in arctic pristine ecosystems with negligible NO^−^_3_ deposition). In these plants, it is also feasible to diagnose leaf NO^−^_3_ reduction using Δ_leaf_ (the net enrichment of NO^−^_3_ isotopes in leaves relative to those of source NO^−^_3_) (Scenarios 3–6; Figure 6).

Scenario 3: If no NO^−^_3_ was transported from soil to leaves, and leaf NO^−^_3_ if any, was completely derived from atmosphere, but no reduction occurred, then:

$$\Delta_{\text{leaf}}=\delta_{\text{leaf}}-\delta_{\text{atm}}\approx 0.$$

Scenario 4: If no NO^−^_3_ was transported from soil to leaves and leaf NO^−^_3_ was acquired from atmosphere; and reduction occurred, then:

$$\Delta_{\text{leaf}}=\delta_{\text{leaf}}-\delta_{\text{atm}}>0.$$

Scenario 5: If all leaf NO^−^_3_ was taken up directly from the soil and no reduction occurred in roots or leaves, then:

$$\Delta_{\text{leaf}}=\delta_{\text{leaf}}-\delta_{\text{soil}}\approx 0.$$

Scenario 6: If leaf NO^−^_3_ was transported completely from the soil and reduction occurred only in the leaves, then

$$\Delta_{\text{leaf}}=\delta_{\text{leaf}}-\delta_{\text{soil}}>0.$$

The induction of NR by atmospheric-derived NO^−^_3_ has been shown in plants exposed to airborne N oxides (e.g., Norby et al., 1989; Wellburn, 1990). Scenarios 3–4 are expected to be true for mosses because atmospheric NO^−^_3_ has been assumed as the sole source (Liu et al., 2012a). Nevertheless, isotopic partitioning of N sources (Liu et al., 2013b) and further Δ^17^O analysis (Figure 5) suggests that moss NO^−^_3_, even at epilithic habitats, is actually a mixture of atmospheric NO^−^_3_ and soil-derived NO^−^_3_. Thus, it is becoming clear that mosses can acquire substantial N from substrates; and moss NO^−^_3_ is a valid atmospheric bio-monitor only for species growing on rare N-free substrates. Scenarios 5–6 demonstrated NO^−^_3_ dynamics of vascular plants in the tundra of northern Alaska, where the Δ^17^O of NO^−^_3_ in plants with surprisingly high [NO^−^_3_] was found as 0‰ (e.g., *Polygonum bistorta*). However, examining only Δ_leaf_ seems insufficient to determine NO^−^_3_ reduction location, since, isotopic enrichments of leaf NO^−^_3_ might result from root reduction activities before moving up to leaves (Scenario 7).

Scenario 7: If the leaf NO^−^_3_ is completely or partly transported from the root where it has experienced reduction, but no reduction has occurred in the leaf; then an isotope mass-balance calculation can be conducted to quantify the amount of leaf NO^−^_3_ accumulated directly from soil and indirectly from roots:

$$\begin{array}{l} \Delta_{\text{root}}=\delta_{\text{leaf}}-\delta_{\text{soil}}\approx \delta_{\text{root}}-\delta_{\text{soil}}>0,\\ \Delta_{\text{leaf}}=\delta_{\text{leaf}}-\delta_{\text{root}}<0,\text{ and}\\ \delta_{\text{leaf}}=(1-f_{\text{root}})\times \delta_{\text{soil}}+f_{\text{root}}\times \delta_{\text{root}}. \end{array}$$

The reduction of NO^−^_3_ that has experienced reduction in roots can further increase the isotopic enrichment of leaf NO^−^_3_ relative to soil NO^−^_3_ (Scenario 8) (Figure 6). This has been demonstrated by the δ^15^N difference between roots and leaves in plants growing with NO^−^_3_ with known δ^15^N values (Yoneyama and Kaneko, 1989; Evans et al., 1996; Yoneyama et al., 2001). This NO_3_ reduction occurs especially in plants that are capable of reducing NO^−^_3_ in both shoots and roots (Stewart et al., 1992).

Scenario 8: If the leaf NO^−^_3_ is completely or partially transported from roots where it has experienced reduction; and if it is further reduced in the leaf. In this case, a partitioning similar to scenario 7 can be done by considering the Δ_leaf_ in the isotope mass-balance calculation:

$$\begin{array}{l} \Delta_{\text{root}}=\delta_{\text{root}}-\delta_{\text{soil}}>0\text{ and}\\ \delta_{\text{leaf}}=[(1-f_{\text{root}})\times \delta_{\text{soil}}+f_{\text{root}}\times \delta_{\text{root}}]+\Delta_{\text{leaf}}. \end{array}$$

Plant NO^−^_3_ in scenarios 1–8 was derived either from the soil or atmosphere (Figure 6). A supplemental diagnosis of NR dynamics was to examine the covariance of Δδ^18^O:Δδ^15^N ratios (Δ is the isotopic enrichment of plant NO^−^_3_ relative to source NO^−^_3_; Δ = δ_plant_ − δ_source_). This diagnosis helped determine whether the N–O bond breakage attributable to NO^−^_3_ reduction was the single process driving NO^−^_3_^15^N and ^18^O enrichments. Theoretically, the dissociation of an O atom from NO^−^_3_ predicted that NO^−^_3_ isotopes would be fractionated in an O-to-N ratio of ca. 0.6 (Brown and Drury, 1967). However, the NR often had the same O-to-N isotopic imprint on substrate NO^−^_3_ in experimental studies. Consequently, the 1:1 trend was considered ubiquitous for biological NO^−^_3_ reduction (Granger et al., 2004, 2010). However, for leaves of vascular plants that acquire NO^−^_3_ from both atmosphere and soil, it is difficult to constrain leaf NO^−^_3_ reduction based only on the Δ_leaf_ (δ_leaf_ − δ_source_) and ε_NR_, because the mixing of atmospheric NO^−^_3_ can raise the δ values (especially δ^18^O). Liu et al. (2013a) observed that the δ^18^O:δ^15^N ratios in roots of a conifer generally followed the 1:1 rule; although leaf NO^−^_3_ showed distinctly higher δ^18^O:δ^15^N ratios (2.5:1) because of the mixing of atmospheric NO^−^_3_.

As described above, the fraction of atmospheric-derived NO^−^_3_ (*F*_atm_) in leaves can be estimated using Δ^17^O mass-balance calculation (*F*_atm_ = Δ^17^O_leaf_ / Δ^17^O_atm_ < 1). Thereafter, the leaf NO^−^_3_ sources and NR dynamics can be further constrained.

Scenario 9: If leaf NO^−^_3_ was absorbed from both the atmosphere and soil, but no reduction occurred in the leaf, then the fraction of atmospheric-derived NO^−^_3_ calculated using δ^18^O or δ^15^N (*f*_atm_) is expected to be similar to *F*_atm_, as

$$\begin{array}{l} \delta_{\text{leaf}}=(1-f_{\text{atm}})\times \delta_{\text{soil}}+f_{\text{atm}}\times \delta_{\text{atm}},\\ \text{and }f_{atm}\approx F_{atm}<1. \end{array}$$

Scenario 10: If leaf NO^−^_3_ was absorbed from both the atmosphere and soil, and reduction occurred in the leaf, then:

$$\begin{array}{l} \delta_{\text{leaf}}=[(1-f_{\text{atm}})\times \delta_{\text{soil}}+f_{\text{atm}}\times \delta_{\text{atm}}]+\Delta_{\text{leaf}},\\ f_{\text{atm}}\approx F_{\text{atm}}<1,\\ \text{and }\Delta_{\text{leaf}}=\delta_{\text{leaf}}-[(1-F_{\text{atm}})\times \delta_{\text{soil}}+F_{\text{atm}}\times \delta_{\text{atm}}]>0. \end{array}$$

Scenario 11: If leaf NO^−^_3_ is a mixture of atm-NO^−^_3_ and root NO^−^_3_, but no reduction occurred, then:

$$\begin{array}{l} \delta_{\text{leaf}}=(1-f_{\text{atm}})\times \delta_{\text{root}}+f_{\text{atm}}\times \delta_{\text{atm}}\\ \;\approx [(1-f_{\text{atm}})\times (\delta_{\text{soil}}+\Delta_{\text{root}})+f_{\text{atm}}\times \delta_{\text{atm}}],\\ f_{\text{atm}}\approx F_{\text{atm}}<1,\\ \text{and }\Delta_{\text{root}}=\delta_{\text{root}}-\delta_{\text{soil}}\\ \;\approx [(\delta_{\text{leaf}}-F_{\text{atm}}\times \delta_{\text{atm}})/(1-F_{\text{atm}})]-\delta_{\text{soil}}>0. \end{array}$$

Scenario 12: If leaf NO^−^_3_ is a mixture of atm-NO^−^_3_ and root NO^−^_3_; and if the reduction occurred in the leaf, then:

$$\begin{array}{l} \delta_{\text{leaf}}=[(1-f_{\text{atm}})\times \delta_{\text{root}}+f_{\text{atm}}\times \delta_{\text{atm}}]+\Delta_{\text{leaf}}\\ \;\approx [(1-f_{\text{atm}})\times (\delta_{\text{soil}}+\Delta_{\text{root}})+f_{\text{atm}}\times \delta_{\text{atm}}]+\Delta_{\text{leaf}},\\ f_{\text{atm}}\approx F_{\text{atm}}<1,\\ \Delta_{\text{root}}=\delta_{\text{root}}-\delta_{\text{soil}}>0,\\ \text{and }\Delta_{\text{leaf}}=\delta_{\text{leaf}}-[(1-f_{\text{atm}})\times \delta_{\text{root}}+f_{\text{atm}}\times \delta_{\text{atm}}]>0. \end{array}$$

Scenario 13: If leaf NO^−^_3_ is a mixture of soil NO^−^_3_, atm-NO^−^_3_, and root NO^−^_3_, but no reduction occurred in the leaf, then:

$$\begin{array}{l} \delta_{\text{leaf}}=(1-f_{\text{atm}}-f_{\text{soil}})\times \delta_{\text{root}}+f_{\text{atm}}\times \delta_{\text{atm}}+f_{\text{soil}}\times \delta_{soil},\\ f_{\text{atm}}\approx F_{\text{atm}}<1,\\ \text{and }\Delta_{\text{root}}=\delta_{\text{root}}-\delta_{\text{soil}}>0. \end{array}$$

Scenario 14: If leaf NO^−^_3_ is a mixture of soil NO^−^_3_, atm-NO^−^_3_, and root NO^−^_3_, and if reduction occurred in the leaf, then:

$$\begin{array}{l} \delta_{\text{leaf}}=[(1-f_{\text{atm}}-f_{\text{soil}})\times \delta_{\text{root}}+f_{\text{atm}}\times \delta_{\text{atm}}\\ \;+f_{\text{soil}}\times \delta_{\text{soil}}]+\Delta_{\text{leaf}},\\ f_{\text{atm}}\approx F_{\text{atm}}<1,\\ \Delta_{\text{root}}=\delta_{\text{root}}-\delta_{\text{soil}}>0,\\ \text{and }\Delta_{\text{leaf}}=\delta_{\text{leaf}}-[(1-f_{\text{atm}}-f_{\text{soil}})\times \delta_{\text{root}}\\ \;+f_{\text{atm}}\times \delta_{\text{atm}}+f_{\text{soil}}\times \delta_{\text{soil}}]>0 \end{array}$$

The parameters in the scenarios 9–14 (*f*_atm_, *F*_atm_, Δ_root_, Δ_leaf_) above, provide theoretical constraints on possible NO^−^_3_ sources and reduction dynamics in leaves of field plants. As explained above, δ^15^N values of NO^−^_3_ often overlapped for soil and atmospheric sources, although δ^18^O and or Δ^17^O can provide a clear differentiation between them (Kendall et al., 2007; Michalski, 2010). Consequently, the scenarios above are better suited to the δ^18^O (depicted in Figure 6) than δ^15^N analysis, particularly when leaf NO^−^_3_ was a mixing pool for different sources. The other solution to diagnose atmospheric NO^−^_3_ mixing and reduction is the Δ^17^O-δ^18^O correlation, which has been used to trace NO^−^_3_ sources and dynamics in aquatic environments (Tsunogai et al., 2011). Although preliminary, the Δ^17^O values in mosses showed clearly higher *F*_atm_ than vascular plants, especially in epilithic mosses. Although, the Δ^17^O in terricolous mosses and vascular leaf samples was as low as 0.0–2.2‰, even at high NO^−^_3_ concentration levels (Figure 5), suggesting a 0.0–8.8% of atmospheric contribution to leaf NO^−^_3_ pool. The NRA should be responsible for δ^18^O enrichment relative to the mixing values if plant-absorbed NO^−^_3_ has not been influenced by denitrification in soil. Such characterization cannot be warranted by correlation between δ^15^N and δ^18^O, or between tissue [NO^−^_3_] and isotopes (e.g., Liu et al., 2012c).

## Uncertainties in tissue NO^−^_3_-isotope methods and future works

Although, the sampling time of plant materials can be controlled, diurnal and seasonal variations in tissue NO^−^_3_ and its isotopes should be verified in future works. Until now, no experimental work has directly examined NR enzymatic isotope kinetics in roots and leaves of higher plants. Moreover, it is difficult to mimic *in situ* NR isotope effects in field conditions. Isotope effects associated with NO^−^_3_ uptake and efflux remain unverified for roots. They were measured recently as 1–3‰ in growing cells of marine diatoms, and different O and N fractionations for both uptake and efflux were thought to cause the net ^18^ε:^15^ε of NO^−^_3_ assimilation above 1 (Karsh et al., 2014). The routes of transformation and entry of inorganic and organic NO^−^_3_ sources from the atmosphere into leaf cells and subsequent cellular actions have not been clarified, especially for non-aqueous processes. Consequently, the sources and supply rates of atmospheric NO^−^_3_ and their isotope signals should be explored further. Thus far, the Δ^17^O information of leaf NO^−^_3_ was sparse, and is mostly available for leaves with high NO^−^_3_ levels. It should be verified whether the atmospheric contribution is higher in low-[NO^−^_3_] leaves or not. It is promising to measure NO^−^_3_ isotopes in xylem flow and twig samples for NO^−^_3_ transportation and translocation. Results of such studies can potentially provide useful insights into intraplant NO^−^_3_ transportation and translocation, although the sampling methods of xylem flow are mostly destructive and in-twig NO^−^_3_ might be very low. For these reasons, more field works on tissue NO^−^_3_ at the organ, stand, and species levels should be done along with source isotope analysis. The scenarios proposed above provide the first conceptual constraint for both sources and NO^−^_3_ isotope effects in field plants. In conclusion, the concentration and isotopic analyses of NO^−^_3_ in plant tissues together provide new insights for elucidating plant NO^−^_3_ sources and strategies. These strategies will be valuable for exploring the communication of plant N utilization with environmental N pollution and altering ecosystem N cycles.

### Conflict of interest statement

The authors declare that the research was conducted in the absence of any commercial or financial relationships that could be construed as a potential conflict of interest.
